# Data on the efficacy of *Echinococcus multilocularis* cryopreservation and subsequent protoscolex development in cotton rats and infectivity in dogs

**DOI:** 10.1016/j.dib.2026.113084

**Published:** 2026-07-18

**Authors:** Hirokazu Kouguchi, Tomohito Koyano, Naoki Hayashi, Masahito Hidaka, Hiroyuki Matsuyama, Shigehiro Enkai, Takeshi Aiyama, Tatsuhiko Kakisaka, Akinobu Taketomi, Ryo Nakao, Nariaki Nonaka

**Affiliations:** a Department of Infectious Diseases, Hokkaido Institute of Public Health, N19 W12, Kita-Ku, Sapporo, Hokkaido, 060-0819, Japan; b Laboratory of Parasitology, Department of Disease Control, Graduate School of Infectious Diseases, Faculty of Veterinary Medicine, Hokkaido University, N18 W9, Kita-ku, Sapporo, Hokkaido, 060-0818, Japan; c Department of Gastroenterological Surgery I, Hokkaido University Graduate School of Medicine, N15 W7, Kita-ku, Sapporo, Hokkaido, 060-0815, Japan; d Division of Parasitology, Veterinary Research Unit, International Institute for Zoonosis Control, Hokkaido University, Sapporo, 001-0020, Japan; e Department of Pediatrics, Teikyo University School of Medicine, 2-11-1 Kaga, Itabashi-ku, Tokyo, 173-8605, Japan; f One Health Research Center, Hokkaido University, N18 W9, Kita-ku, Sapporo, Hokkaido, 060-0818, Japan

**Keywords:** *Echinococcus multilocularis*, Echinococcosis, Cotton rat, Cryopreservation, Passage, Preservation, Freeze

## Abstract

This dataset presents information on the viability of cryopreserved *Echinococcus multilocularis* (the Nemuro strain, Alaskan lineage) stored in liquid nitrogen. Protoscoleces and cyst residues were obtained from cotton rats experimentally infected with parasite eggs in the laboratory. These materials were mixed with a sperm cryopreservation solution and frozen in liquid nitrogen for one month. The viability of the cryopreserved material was evaluated by intraperitoneal injection into cotton rats, follows by the observation of cyst and protoscolex development within the peritoneal cavity at four months post-infection. The experiment was conducted independently on three separate occasions using two *E. multilocularis* strains (Nemuro and European strains). In total, *E. multilocularis* cysts were detected in 18 of the 20 inoculated cotton rats. Furthermore, the development of protoscoleces was observed in the cysts that developed in all animals. The protoscoleces purified from the cysts formed within the peritoneal cavity of a cotton rat were administered orally to a beagle dog, and the presence of parasite eggs in the feces was monitored. Consequently, parasite eggs were detected in the feces between days 30–35 post-infection. This confirms that the protoscoleces obtained from cryopreserved material can serve as an infection source for the definitive host. The methods described in this study will allow for the simple maintenance of *E. multilocularis* strains with minimal animal use and reduced costs.

## Graphical abstract

**Figure:**
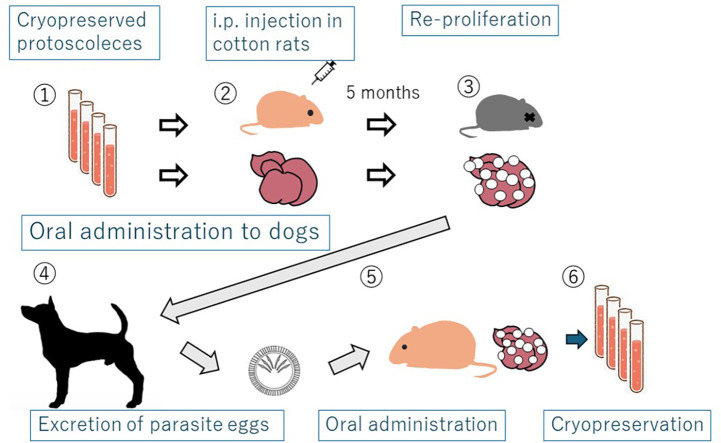

Specifications Table

**Table:** 

Subject	Biology
Specific subject area	Cryopreservation of *Echinococcus multilocularis* strains
Type of data	Table, Figure
Data collection	Viability of cryopreserved protoscoleces and cyst residues was examined evaluated by intraperitoneal injection into cotton rats, follows by the observation of cyst and protoscolex development within the peritoneal cavity at four months post-infection. The protoscoleces purified from the cysts formed within the peritoneal cavity of a cotton rat were administered orally to a beagle dog, and the presence of parasite eggs in the feces was monitored.
Data source location	Institution: Hokkaido Institute of Public Health City: Sapporo Country: Japan Latitude and longitude (and GPS coordinates) for collected samples/data: 43 °04′58.804′'N; 141 °19′59.769′'E.
Data accessibility	Repository name: Data on the regrowth of cryopreserved E. multilocularis larvae in cotton rats and their infectivity to dogs Data identification number: doi: 10.17632/nsdsvc7g8p.1 Direct URL to data: https://data.mendeley.com/datasets/nsdsvc7g8p/1
Related research article	none

## 1 Value of the Data

• This dataset presents qualitative and quantitative data on a reliable cryopreservation method for *E. multilocularis* strains.
• At present, the primary method for maintaining *E. multilocularis* strains involves repeated intraperitoneal injections of parasite material into experimental animals, requiring new animals for each strain every 3 to 6 months. These data are valuable because the reported methods can contribute to reduction of experimental animals required for the maintenance of *E. multilocularis* strains, thereby supporting the ethical and sustainable use of animals in biomedical research.
• These data demonstrate that protoscoleces obtained from the cryopreserved material maintain their infectivity towards the definitive host (dog).
• The reported methods will assist researchers in maintaining parasite strains with reduced costs and labor, thereby facilitating advanced downstream analyses such as comparative studies between strains.

## 2 Background

The cryopreservation of *Echinococcus multilocularis* has not yet become a standard practice. This is because a failure in the process can result in the loss of valuable parasite material. Consequently, the serial passaging within experimental animals infected with secondary echinococcosis remains the only reliable method for maintaining *E. multilocularis* strains. To date, only a few studies have explored the feasibility of cryopreserving *E. multilocularis*. Notably, Eckert et al. [1,2] preserved the larvae of *E. multilocularis* isolated in Switzerland in liquid nitrogen for one week by utilizing stepwise cooling conditions using glycerol or dimethyl sulfoxide as cryoprotectants. They demonstrated that the larvae retained their ability to grow within the peritoneal cavity of gerbils. Additionally, Bretagne et al. [3] successfully stored multiple isolates in 10% glycerol using controlled cooling at 1 °C/min. More recently, Laurimäe et al. [4] confirmed that approximately 40% of the metacestodes cryopreserved in the 1980s remained viable after 35 years, demonstrating the potential of long-term storage in liquid nitrogen. Accordingly, we collected data to investigate whether these methods could be successfully applied to isolates originating from Hokkaido, Japan, and whether the cryopreserved protoscoleces retained their infectivity toward the definitive host.

## 3 Data Description

### 3.1 Cryopreservation samples

**Fig. 1:**
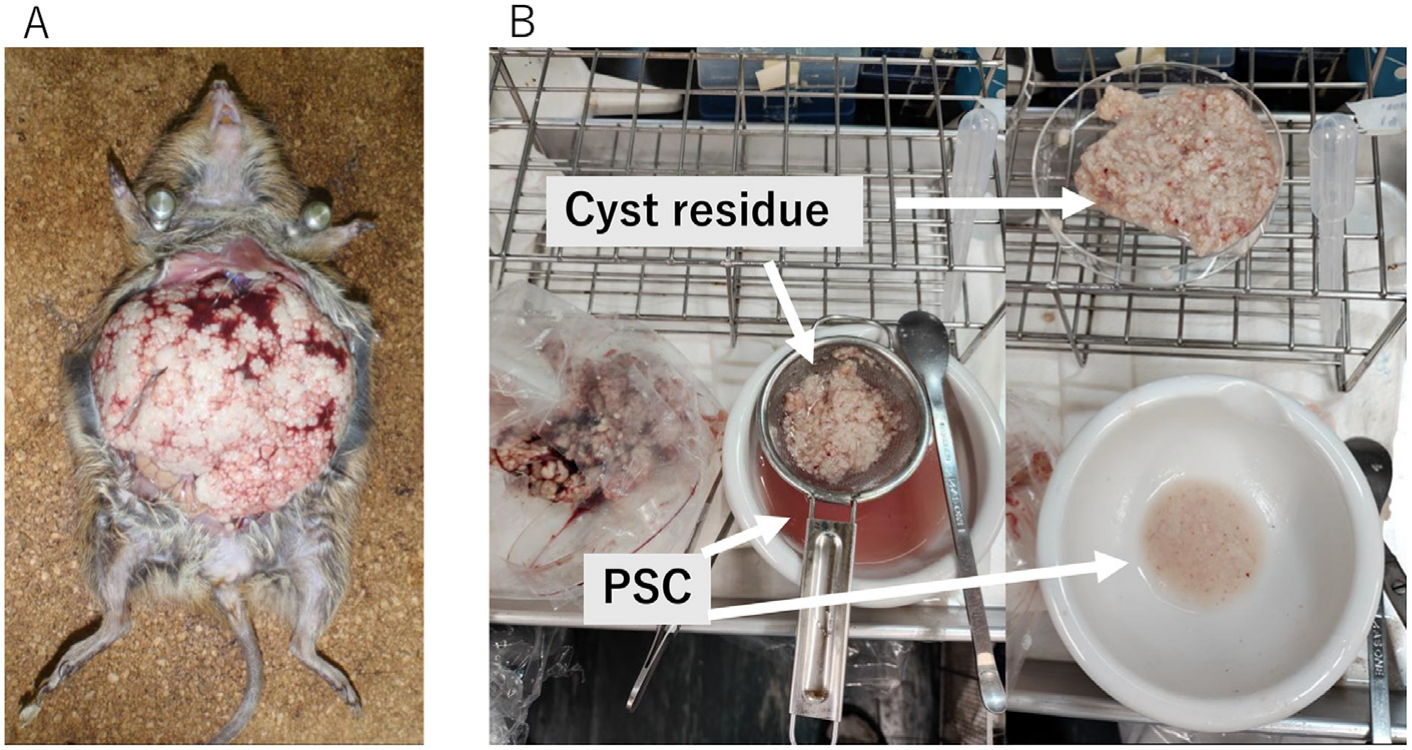
(A) A cotton rat (primary echinococcosis) that was orally administered 200 parasite eggs. (B) Preparation of protoscoleces (PSCs) and cyst residue from primary echinococcal cysts. Cysts extracted from infected cotton rats were minced inside a tea strainer and crushed with a spatula. The material remaining in the strainer was designated as cyst residues, while the filtrate was designated as the PSCs.

Fig. 1A shows a cotton rat experimentally infected with *E. multiloculari*s. Parasite eggs were administered orally to an adult cotton rat, and a necropsy was conducted four months post-infection. Microcysts consisting of clusters of sac-like structures, the typical appearance of the metacestode stage of *E. multilocularis*, were observed. Fig. 1B shows the protoscoleces and cyst residues obtained from the primary microcysts shown in Fig. 1A to serve as cryopreservation material.

### 3.2 Efficacy of cryopreserved samples

**Fig. 2:**
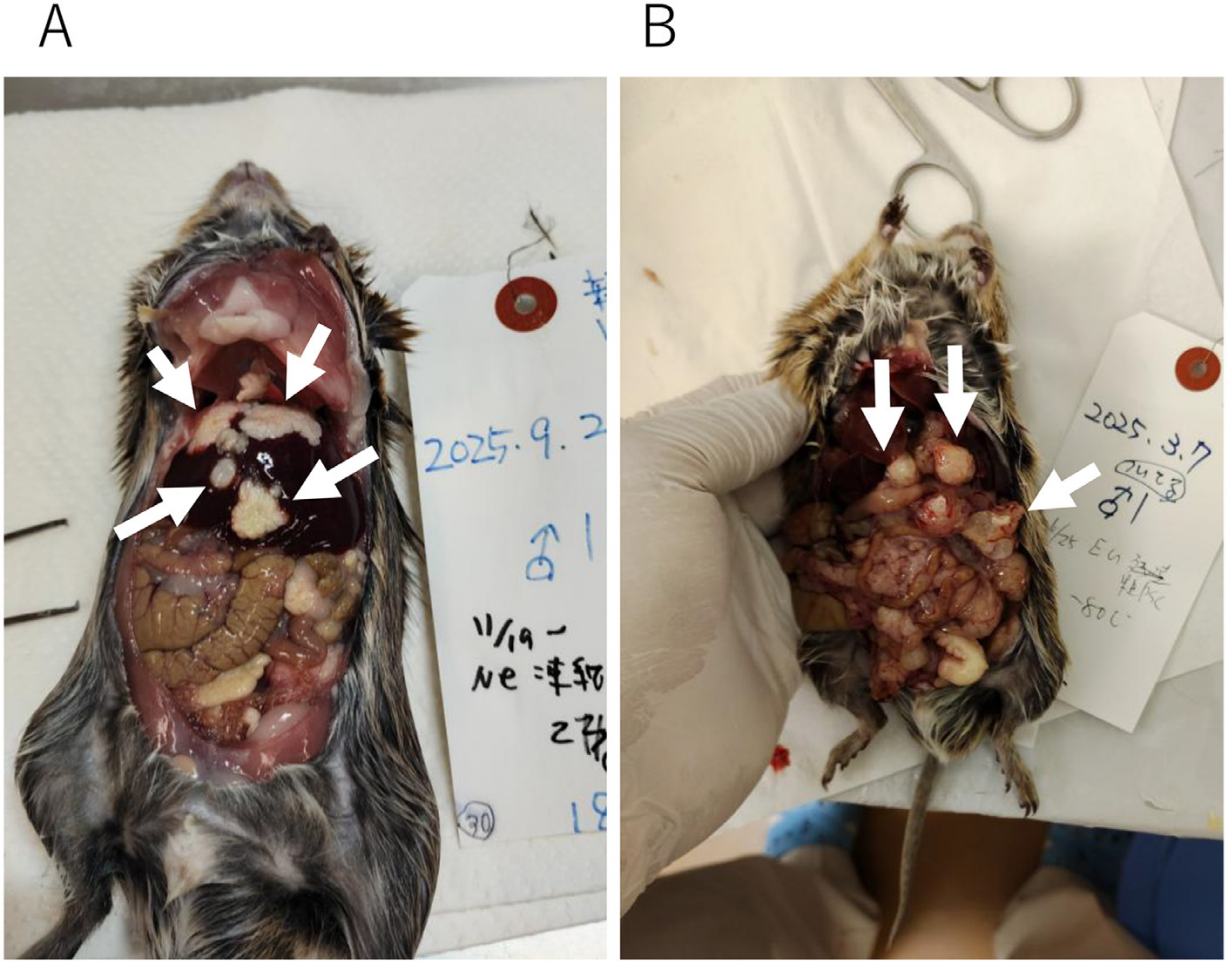
Observation of *E. multilocularis* cysts in cotton rats intraperitoneally injected with the cryopreserved material obtained from both the Nemuro (A) and European (B) strains. Arrows indicate the developed cysts.

Fig. 2 shows the cysts that developed following the intraperitoneal injection of the cryopreserved material obtained from both the Nemuro (A) and European (B) strains.

Table 1 summarizes the results of the intraperitoneal injection of cryopreserved parasite material in cotton rats. In total, *E. multilocularis* cysts were detected in 18 of the 20 inoculated cotton rats. The success rates for protoscoleces and cyst residue were 13 out of 13 and 5 out of 7, respectively. In the first experiment, infection was confirmed in four of the five cotton rats inoculated with the Nemuro strain and four of the five cotton rats inoculated with the European strain. The two animals that did not develop cysts had been injected with cryopreserved cyst residues. In the second and third experiments, *E. multilocularis* cysts were observed in all 10 cotton rats injected with the parasite material, including those receiving cyst residues. Protoscoleces were observed in the cysts that developed in all animals.

**Table 1:** Results of animal experiments evaluating the viability of cryopreserved *Echinococcus multilocularis* based on protoscolex development in cotton rats and infectivity in a dog.

			Results		
Experiment no.	Strain	Material	Presence of cysts	Presence of protoscoleces	Infectivity in dog
First	Nemuro	PSCs	yes	yes	
		PSCs	yes	yes	
		Cyst residues	no	―	
		Cyst residues	yes	yes	
		Cyst residues	yes	yes	
	European	PSCs	yes	yes	
		PSCs	yes	yes	
		PSCs	yes	yes	
		Cyst residues	no	―	
		Cyst residues	yes	yes	
Second	Nemuro	PSCs	yes	yes	yes
		PSCs	yes	yes	
		Cyst residues	yes	yes	
		Cyst residues	yes	yes	
Third	Nemuro	PSCs	yes	yes	
		PSCs	yes	yes	
		PSCs	yes	yes	
		PSCs	yes	yes	
		PSCs	yes	yes	
		PSCs	yes	yes	

PSCs, protoscoleces.

The protoscoleces purified from the cysts formed in the peritoneal cavity of a cotton rat in the second experiment were administered orally to a beagle dog. The presence of parasite eggs in the feces of the dog was monitored every other day from day 28 of post-infection. Consequently, a large number of eggs were detected in the feces between days 30–35 post-infection. Egg per gram (epg) at days 30 through 35 post-infections were 86.9, 2640, 3098, 1653, 98.0, and 381, respectively. This confirms that the protoscoleces obtained from cryopreserved material can serve as an infection source for the definitive host.

## 4 Experimental Design, Materials, and Methods

### 4.1 Strains

Two strains of *E. multilocularis*, the Nemuro and European strains, were used in this study. The Nemuro strain was isolated in 1987 from an infected wild vole (*Myodes rufocanus bedfordiae*) in the Nemuro region (Hokkaido, northern island of Japan) [5]. It belongs to the Alaska lineage [6]. The European strain was provided by Dr. Auer (University of Vienna, Austria) in 1989 [5]. For >30 years, these strains have been maintained through secondary echinococcosis using cotton rats and/or a dog/cotton rat cycle within the BSL3 facility at the Hokkaido Institute of Public Health, Japan.

### 4.2 Cryopreservation of *E. multilocularis*

The infection of dogs, purification of protoscoleces, and infection of cotton rats were performed as previously reported [7]. To obtain parasite material for cryopreservation, cysts harvested from cotton rats orally inoculated with parasite eggs were minced using scissors inside a tea strainer (approximately 0.5 mm mesh, single-layered). While washing with phosphate-buffered saline containing penicillin G (1000,000 units/L; Meiji Seika Pharma Co., Ltd., Tokyo, Japan) and 1 g/L of streptomycin (Meiji Seika Pharma Co., Ltd.), the extracted cysts were crushed with a laboratory spatula and passed through a strainer to isolate the protoscoleces. The material remaining in the strainer was collected for use as cyst residues in further experiments. After allowing each sample to stand for 10 min, the supernatant was completely discarded. Approximately 20 mL of precipitated protoscoleces obtained from the cyst were divided into 15 mL plastic tubes, with 2 mL in each tube. Identical procedure was performed on the cyst residue. Thereafter, 0.67 mL (3:1) of Arctic™ Sperm Cryopreservation Medium (No. 90170, FUJIFILM Irvine Scientific, Inc., USA) was gently added dropwise to reach a final glycerol concentration of 7%, and the tubes were thoroughly inverted to mix the contents. The samples were then pre-cooled at 4 °C for 60 min and at –80 °C for 30 min, followed by storage in liquid nitrogen for at least one month.

### 4.3 Animal experiments

Cryopreserved samples were thawed for 10 min at room temperature and the for 10 min in a water bath at 37 °C. Approximately 2 mL of the 2.67 mL thawed solution was inoculated into the peritoneal cavity of cotton rats (4–16 weeks old, male or female) using an 18-gauge syringe under light anesthesia via isoflurane. Frozen protoscoleces and cyst residues obtained from the Nemuro and European strains were inoculated into 2–6 cotton rats. Four months post-inoculation, the cotton rats were euthanized under deep isoflurane anesthesia and necropsied.

For the Nemuro strain, three independent infection experiments were conducted using cotton rats. Protoscoleces were purified from the extracted cysts using the aforementioned method. Thereafter, an aliquot was transferred to a 24-well plate and observed under a microscope. To determine whether the protoscoleces obtained from cysts that developed in animals inoculated with cryopreserved material remained infectious to the definitive host, 500,000 protoscoleces were orally administered to a beagle dog (female, four months old). Five weeks after administration of protoscoleces, the infectivity in the definitive host was assessed by detecting eggs in the feces using the direct smear method. The number of parasite eggs in the feces was determined by the sucrose flotation method reported previously [7]

## Limitations

Not applicable.

## Ethics Statement

This study was performed in strict accordance with the National Institutes of Health Guide for the Care and Use of Laboratory Animals. The ethics committee of the Hokkaido Institute of Public Health approved the protocol for the animal experiments (permit numbers: K25-2 and K22-1). All surgeries were performed under sodium pentobarbital anesthesia, and every effort was made to minimize suffering.

## CRediT Author Statement

Hirokazu Kouguchi: Conceptualization, Investigation, and Writing - original draft. Tomohito Koyano: Validation. Naoki Hayashi: Validation. Masahito Hidaka: Writing - review & editing. Hiroyuki Matsuyma: Writing - review & editing. Shigehiro Enkai: Funding acquisition. Takeshi Aiyama: Supervision. Tatsuhiko Kakisaka: Supervision. Akinobu Taketomi Supervision. Ryo Nakao: Project administration. Nariaki Nonaka: Supervision and Funding acquisition.

## Data Availability

Mendeley DataData on the regrowth of cryopreserved E. multilocularis larvae in cotton rats and their infectivity to dogs (Original data)
